# Targeting Cytotoxin-Associated Antigen A, a Virulent Factor of *Helicobacter pylori*-Associated Gastric Cancer: Structure-Based In Silico Screening of Natural Compounds

**DOI:** 10.3390/molecules27030732

**Published:** 2022-01-23

**Authors:** Shan He, Abdulraheem Ali Almalki, Misbahuddin M. Rafeeq, Ziaullah M. Sain, Amany I. Alqosaibi, Mashael M. Alnamshan, Ibtesam S. Al-Dhuayan, Abdul Rahaman, Yang Zhang, Hamsa Jameel Banjer, Farah Anjum, Haitham Ali M. Alzghaibi, Ali H. Alharbi, Qazi Mohammad Sajid Jamal

**Affiliations:** 1 School of Chemistry and Chemical Engineering, Guangzhou University, Guangzhou 510006, China; 2112005093@e.gzhu.edu.cn; 2 Institute for Nano Scale Science and Technology, College of Science and Engineering, Flinders University, Bedford Park, SA 5042, Australia; 3 Department of Clinical Laboratory Sciences, College of Applied Medical Sciences, Taif University, P.O. Box 11099, Taif 21944, Saudi Arabia; almalki@tu.edu.sa (A.A.A.); h.banjer@tu.edu.sa (H.J.B.); 4 Department of Pharmacology, Faculty of Medicine, King Abduaziz University, Jeddah 21589, Saudi Arabia; marafeeq@kau.edu.sa; 5 Department of Microbiology, Faculty of Medicine, King Abduaziz University, Jeddah 21589, Saudi Arabia; zsain@kau.edu.sa; 6 Department of Biology, College of Science, Imam Abdulrahman Bin Faisal University, P.O. Box 1982, Dammam 31441, Saudi Arabia; amgosaibi@iau.edu.sa (A.I.A.); malnamshan@iau.edu.sa (M.M.A.); ialdhuayan@iau.edu.sa (I.S.A.-D.); 7 School of Food Science and Engineering, South China University of Technology, Guangzhou 510641, China; rahaman_knabdul@ymail.com; 8 Department of Health Informatics, College of Public Health and Health Informatics, Qassim University, Al Bukayriyah 52741, Saudi Arabia; halzghaibi@qu.edu.sa (H.A.M.A.); ahhrbie@qu.edu.sa (A.H.A.)

**Keywords:** gastric cancer, CagA, natural compounds, virtual screening

## Abstract

Gastric cancer is the fifth most frequent cancer and the third major cause of mortality worldwide. *Helicobacter pylori*, a bacterial infection linked with GC, injects the cytotoxin-associated antigen A (CagA; an oncoprotein) into host cells. When the phosphorylated CagA protein enters the cell, it attaches to other cellular components, interfering with normal cellular signaling pathways. CagA plays an important role in the progression of GC by interacting with phosphatidylserine of the host cell membrane. Therefore, disrupting the CagA–phosphatidylserine connection using small molecules appears to be a promising therapeutic approach. In this report, we screened the natural compounds from ZINC database against the CagA protein using the bioinformatics tools. Hits were initially chosen based on their physicochemical, absorption, distribution, metabolism, excretion, and toxicity (ADMET) characteristics, as well as other drug-like characteristics. To locate safe and effective hits, the PAINS filter, binding affinities estimation, and interaction analysis were used. Three compounds with high binding affinity and specificity for the CagA binding pocket were discovered. The final hits, ZINC153731, ZINC69482055, and ZINC164387, were found to bind strongly with CagA protein, with binding energies of −11.53, −10.67, and −9.21 kcal/mol, respectively, which were higher than that of the control compound (−7.25 kcal/mol). Further, based on binding affinity and interaction pattern, two leads (ZINC153731, ZINC69482055) were chosen for molecular dynamics (MD) simulation analysis. MD results showed that they displayed stability in their vicinity at 100 ns. This study suggested that these compounds could be used as possible inhibitors of CagA protein in the fight against GC. However, additional benchwork tests are required to validate them as CagA protein inhibitors.

## 1. Introduction

Gastric cancer (GC) is the 5th most prevalent malignancy and the 3rd largest cause of cancer mortality, accounting for about 0.8 million deaths globally in 2018. East Asian nations such as Japan, China, and Korea account for more than half of all GC patients, and the incidence of GC in these countries is about 10 times greater than in the United States [1]. *Helicobacter pylori*, the causal agent of GC, is a Gram-negative microaerophilic bacterium that infects the stomach epithelium [2,3] and has been shown to infect nearly half of the world’s population, making it one of the most prevalent human infectious agents globally [4,5]. 

Bacteria have evolved various methods for secreting proteins or injecting poisons into target cells. *H. pylori* injects the cytotoxin-associated antigen A (CagA; an oncoprotein) into host cells via the cag Type IV secretion system [6]. CagA is the only oncoprotein that has been demonstrated to be transported by *H. pylori*. In terms of delivery, CagA acts as a bacterium-derived scaffolding/adaptor protein inside the host cell, causing gastric mucosa carcinogenesis [7]. Once within the cell, the phosphorylated CagA protein attaches to other cellular molecules, interfering with normal cellular signaling pathways [8]. CagA is particularly effective in disrupting the processes that maintain normal epithelial differentiation, such as cell adhesion, cell polarity, and cell migration inhibition [9]. Given that the contact between the CagA protein and the membrane phosphatidylserine (PS) is essential for CagA protein entrance into the host cell, blocking the interaction with small molecules looks to be a viable therapeutic approach [7,10].

As part of a multidisciplinary drug discovery strategy, computer-aided drug design (CADD) has achieved widespread acceptance among biologists and chemists [11]. CADD is widely employed in the pharmaceutical industry to decrease cost and time and accelerate the early-stage development of physiologically novel active compounds, and it plays an important role in drug discovery, design, and analysis [12]. Phytochemicals are frequently safer and more chemically diverse than synthetic medications derived from commercial sources, and they often have important pharmacological properties such as antibacterial, anticancer, antioxidative, and anti-inflammatory actions [13,14,15]. Hence, phytochemicals are gaining popularity among clinical researchers and gastroenterologists as a means of developing time-effective treatment alternatives for eliminating *H. pylori* infection with negligible side effects [16]. Using bioinformatics approaches, this study aimed to find new potential leads from the ZINC database that could be used to block the CagA-PS binding interaction in order to fight GC. 

## 2. Methodology

### 2.1. Protein Preparation

The crystal structure of CagA protein (PDB id: 4DVZ) was taken from the protein data bank [17]. CagA has a structured N-terminal domain and an inherently disordered C-terminal region that regulates a wide range of protein interactions. N-terminal CagA fragment has three domains (Domain I, II, and III). Domain II is the PS binding domain and transports the CagA protein to the host cell membrane. Leads were docked onto the positively charged helix α18 active site (residues 610–639).

### 2.2. Virtual Screening

Virtual screening was utilized to identify ligands that interact with CagA protein. In this study, natural compounds from a commercially accessible ZINC library were utilized for virtual screening with the PyRx 0.8 program. PyRx was used to prepare the whole ligands before molecular docking to obtain multiple binding conformations and the lowest binding energy (BE).

### 2.3. Molecular Docking

The Autodock4.2 program [18] was used to clarify the binding conformations of hit compounds with the CagA protein. Hits were docked onto the positively charged helix α18 active site (mainly on Arg624 amino acid). The grid center points X, Y, and Z were set as −0.306, 38.831, and −4.786, respectively. Grid points were fixed as 78 × 50 × 97 Å with the spacing of 0.375 Å. Other AutoDock parameters were set to be the default. The conformation with the lowest BE was chosen as the best.

### 2.4. In Silico Physicochemical, Pharmacokinetics, Drug-Likeness and ADMET Prediction

By using molecular modeling techniques to discover innovative drug candidates, the time required for drug development is significantly shortened and the success rate is much enhanced. For the preliminary assessment of physicochemical, pharmacokinetic, and drug-like characteristics in the drug development process, standard computational pharmacokinetics parameters and drug-likeness were created. Three best scoring natural compounds were evaluated for their physicochemical, drug-likeness, and ADMET properties using the pkCSM web server [19] and datawarrior [20] tools.

### 2.5. Molecular Dynamics Simulation

The use of molecular dynamics (MD) simulation to visualize macromolecule flexibility [21] is a useful tool. Many unknown biological activities and complex dynamic processes can be discovered by examining the internal movements of proteins [22,23,24]. GROMACS 5.1.2 [25] was used to perform MD simulations on CagA-free, CagA-ZINC153731, and CagA-ZINC69482055 at 300 K, with the GROMOS96 43a1 force-field [26]. PRODRG server [27] was employed for the generation of topology as well as force-field parameters of the selected ligands. 

CagA-free, CagA-ZINC153731, and CagA-ZINC69482055 were waterlogged in a ‘cubic box’ with a primary diameter of 8 nm and retaining all the default parameters. The system was then minimized using 1500 ‘steepest descent’ steps, and the temperature of all systems was increased from 0 to 300 K over the course of their equilibration time (100 ps), while maintaining a constant volume and periodic boundary conditions.

The equilibration process was divided into two stages: NVT ensemble and NPT ensemble. The original structures’ C backbone atoms were restrained, while all other atoms were free to move in both NVT and NPT. The MD was then performed at 300 K on a time scale of 100 ns. GROMACS analysis modules were used to investigate the resulting trajectories. All graphical representations were created using PyMOL and VMD [28]. 

## 3. Results and Discussion

CagA (an oncoprotein) plays a key role in the progression of GC and has been identified as a therapeutic target in GC prevention [29,30]. This study screened the natural compounds from the ZINC database targeting the CagA protein of *H. pylori*. The selected compounds (ZINC153731, ZINC69482055, and ZINC164387) preserve an acceptable range of physicochemical, pharmacokinetics, drug-likeness, and ADMET attributes, as per computational predictions (Table 1 and Table 2). According to the datawarrior tool’s estimated drug-likeness values, around 80% of marketed drugs do have positive value. However, commercially available chemicals account for the vast majority of negative values. Positive drug-likeness values were found for ZINC153731 and ZINC69482055, showing that these compounds are more likely to be commercial drugs.

The best scoring (−11.53 kcal/mol) compound, ZINC153731, also known as methyl p-hydroxycinnamate, is a methyl ester of hydroxycinnamic acid and has been shown to have anti-tumor, anti-oxidant, anti-adipogenic, and depigmenting properties. Numerous medicinal plants have been reported to contain it, including Clausena harmandiana, Plumeria obtuse, Sorghum bicolor, and Idesia polycarpa [31,32].

**Figure 1:**
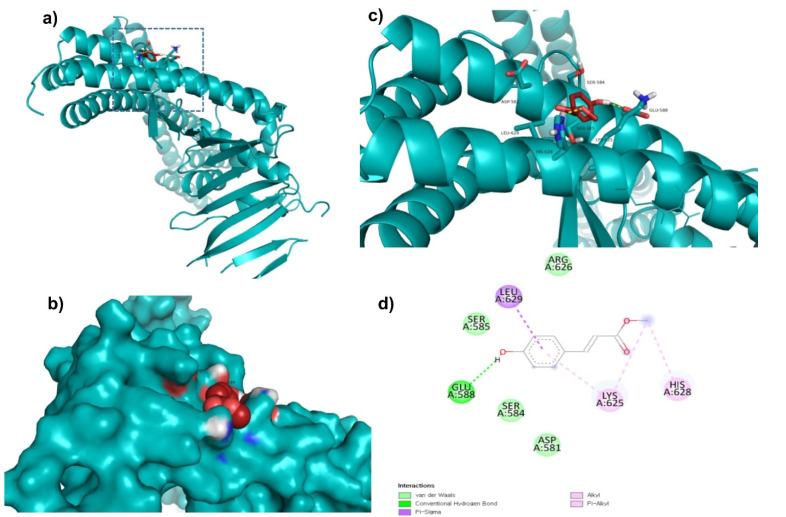
Visualization and surface view of ZINC153731 in the active site of CagA protein (**a**,**b**). Three-dimensional (**c**) and 2D (**d**) view of CagA residue interacting with ZINC153731.

Top lead compounds (ZINC153731, ZINC69482055, and ZINC164387) were found to bind strongly with CagA protein. ZINC153731 was found to interact with Asp581, Ser584, Ser585, Glu588, Lys625, Arg626, His628, and Leu629 amino acid residues of CagA protein (Figure 1). Of these residues, Asp581, Ser584, Ser585, and Arg626 residues of CagA were involved in van der Waals interaction with ZINC153731. BE and inhibition constant for ZINC153731-CagA protein complex were observed to be −11.53 kcal/mol and 10.9 µM, respectively (Table 3).

**Figure 2:**
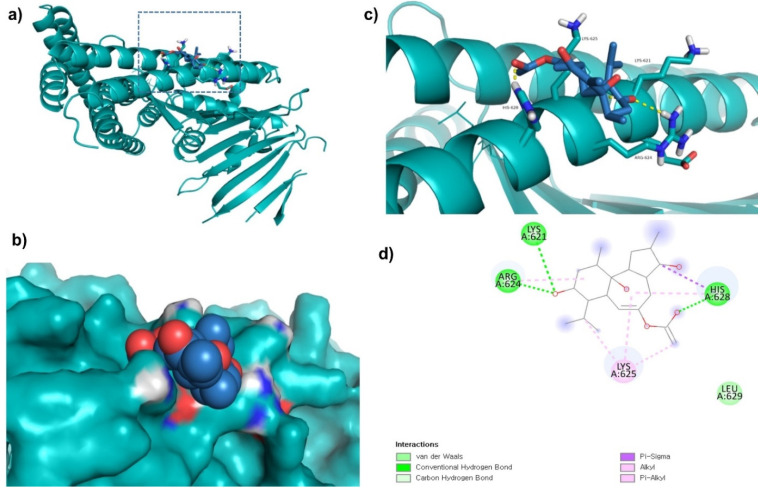
Visualization and surface view of ZINC69482055 in active site of CagA protein (**a**,**b**). Three-dimensional (**c**) and 2D (**d**) view of CagA residue interacting with ZINC69482055.

ZINC69482055 was observed to bind with five amino acid residues (Lys621, Arg624, Lys625, His628, and Leu629) of CagA protein (Figure 2). Leu629 of CagA protein formed van der Waals interaction with ZINC69482055. BE and inhibition constant for ZINC69482055-CagA protein complex were observed to be −10.67 kcal/mol and 13.32 µM, respectively (Table 3).

**Figure 3:**
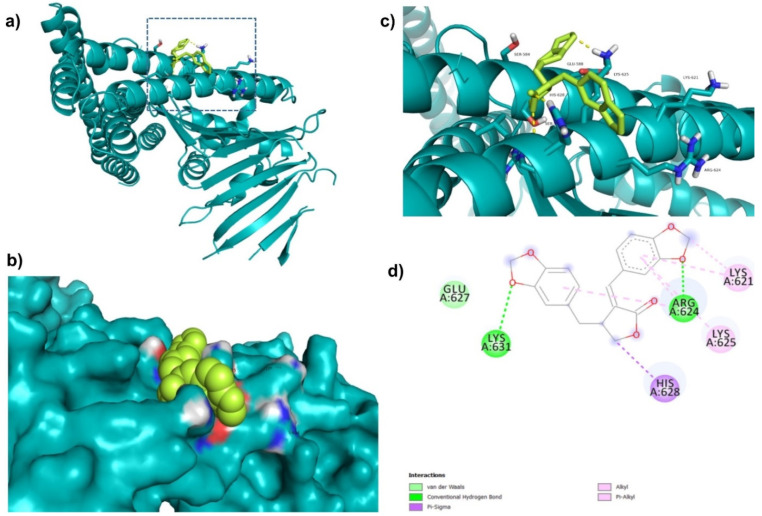
Visualization and surface view of ZINC164387 in active site of CagA protein (**a**,**b**). Three-dimensional (**c**) and 2D (**d**) view of CagA residue interacting with ZINC164387.

Further, ZINC164387 was found to bind with Lys621, Arg624, Lys625, Glu627, His628, and Lys631 residues of CagA protein (Figure 3). Glu627 residue of CagA showed van der Waals interaction with ZINC164387 (Figure 3). BE and inhibition constant for ZINC164387-CagA protein complex were observed to be −9.21 kcal/mol and 18.56 µM, respectively (Table 3).

**Figure 4:**
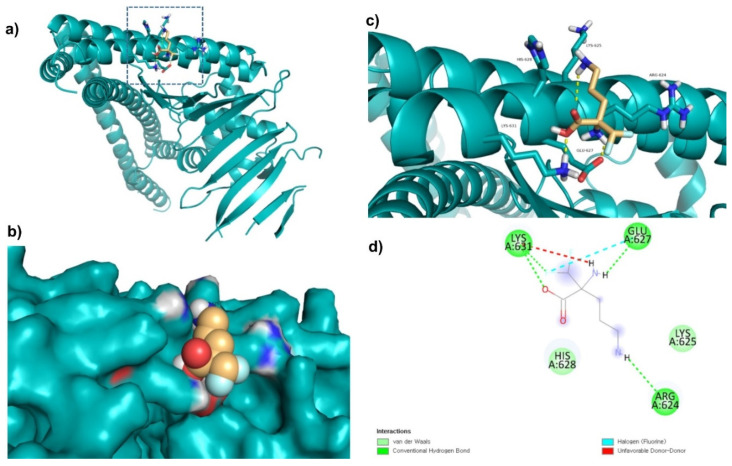
Visualization and surface view of DFMO in active site of CagA protein (**a**,**b**). Three-dimensional (**c**) and 2D (**d**) view of CagA residue interacting with DFMO.

Di-fluoromethylornithine (DFMO) was used as the control compound in this study due to its previously reported inhibitory effect on CagA [33]. DFMO was observed to bind with five amino acid residues (Arg624, Lys625, Leu627, His628, and Lys631) of CagA protein (Figure 4). Lys625 and His628 residues of CagA form van der Waals interaction with DFMO. BE and inhibition constant for DFMO-CagA protein complex were observed to be −7.25 kcal/mol and 28.85 µM, respectively (Table 3). 

When CagA is delivered to gastric epithelial cells, it interacts with numerous molecules in the cells, causing them to become malignant. N-terminal (Domain II) of CagA comprises a basic patch that is important for its inner cell membrane localization and interaction with PS in the plasma membrane of the host cell [34]. Two arginine residues i.e., Arg624 and Arg626 in α-helix of the domain, are crucial for CagA-PS interaction. In addition, it has been revealed that both the arginine residues form a basic amino acid cluster with numerous lysine residues (613, 614, 617, 621, 631, and 635), providing a positive electrostatic surface potential necessary for CagA binding to negatively charged phosphate groups of PS [35]. Hence, inhibiting the CagA-PS interaction is a potential strategy for GC prevention. Interestingly, this study showed that ZINC153731, ZINC69482055, and ZINC164387 interact with the Arg624 residue of CagA, implying that these compounds can disrupt the binding of CagA with the PS of the host cell membrane.

**Figure 5:**
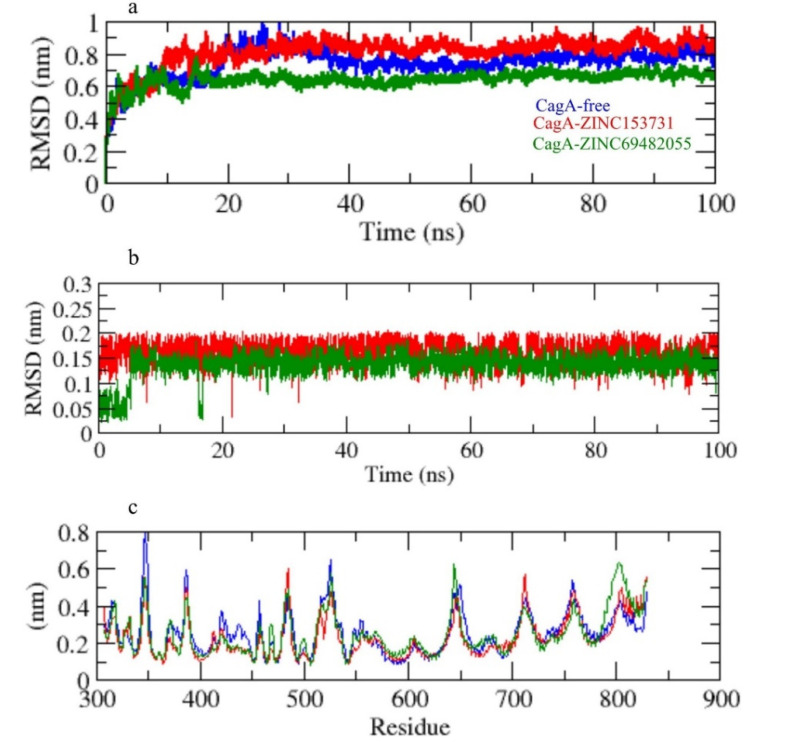
(**a**) RMSD of CagA, (**b**) RMSD of ligand in the pocket, (**c**) RMSF.

The root mean square deviation (RMSD) is a vital fundamental parameter for identifying whether a protein is stable and adheres to its experimental structure [36]. The RMSD average values for CagA-free, CagA-ZINC153731, and CagA-ZINC69482055 were 0.81, 0.84, and 0.62 nm, respectively. The RMSD plot showed that ZINC69482055 binding more effectively stabilized the CagA and resulted in smaller structural deviations from its normal conformation. The CagA-ZINC153731 complex showed a high deviation in the bound structure (Figure 5a). The ligand RMSD also showed that ZINC69482055 binds better than ZINC153731 and is more stable (Figure 5b).

The CagA-free and ZINC153731 backbones displayed continuous fluctuations in the CagA pocket site, most likely due to different orientations, with the largest fluctuation region observed between 340–360 and 790–820 residues (Figure 5c). The vibrations around the equilibrium are not random, but rather depend on the local structure’s flexibility. The root mean square fluctuation (RMSF) of CagA upon binding with ZINC153731 and ZINC69482055 was exhibited as a function of residue numbers to CagA, as well as the average fluctuation of all residues during the simulation. The RMSF plot indicated that CagA had residual variations in multiple protein domain areas. ZINC69482055 and ZINC153731 have been demonstrated to minimize the residual fluctuations.

**Figure 6:**
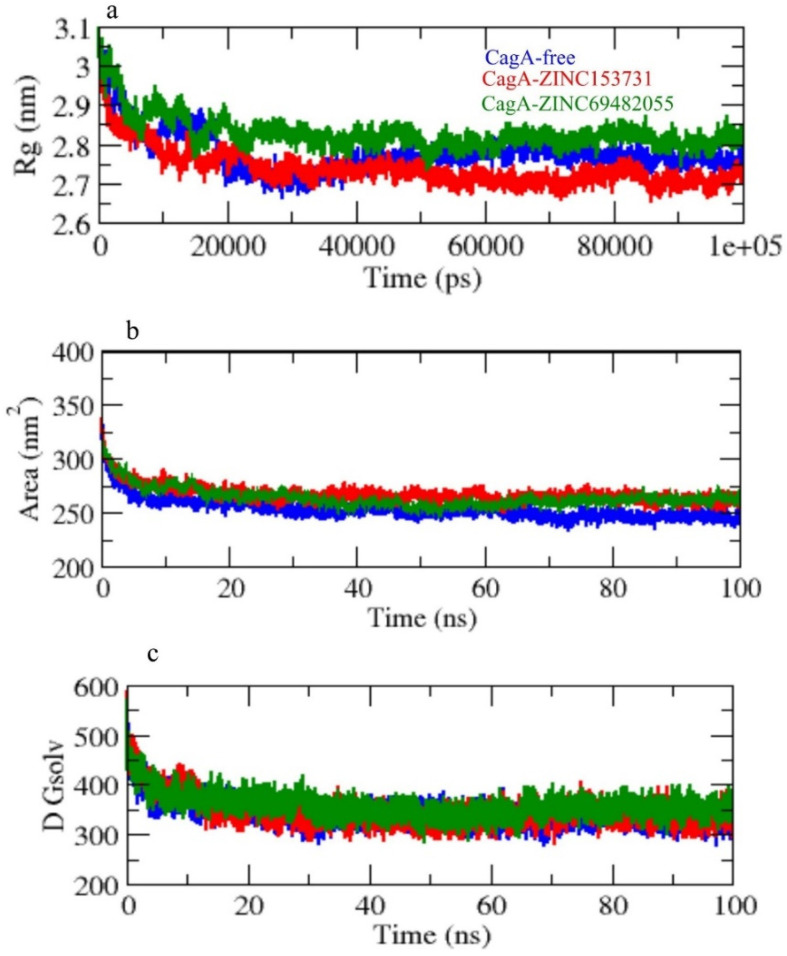
(**a**) Rg, (**b**) SASA, and (**c**) solvent free energy.

Radius of gyration (Rg) was employed to investigate the stability of the protein in a biological system. Because of less-compact packing, a protein should have a wider radius of gyration. CagA-free, CagA-ZINC153731, and CagA-ZINC69482055 had average Rg values of 2.75, 2.70, and 2.85 nm, respectively. The Rg plot showed that CagA achieved tighter packing without the hits and less packing with complex in CagA-ZINC153731, and CagA-ZINC69482055 (Figure 6a).

Solvent accessible surface area (SASA) refers to the region of a protein’s surface that interacts with its solvent molecules [37]. Average SASA values for CagA-free, CagA-ZINC153731, and CagA-ZINC69482055 complexes were observed throughout the 100 ns scale simulation. CagA-free, CagA-ZINC153731, and CagA-ZINC69482055 complexes had average SASA values of 255.01, 280.61, and 265.21 nm^2^, respectively (Figure 6b,c).

**Figure 7:**
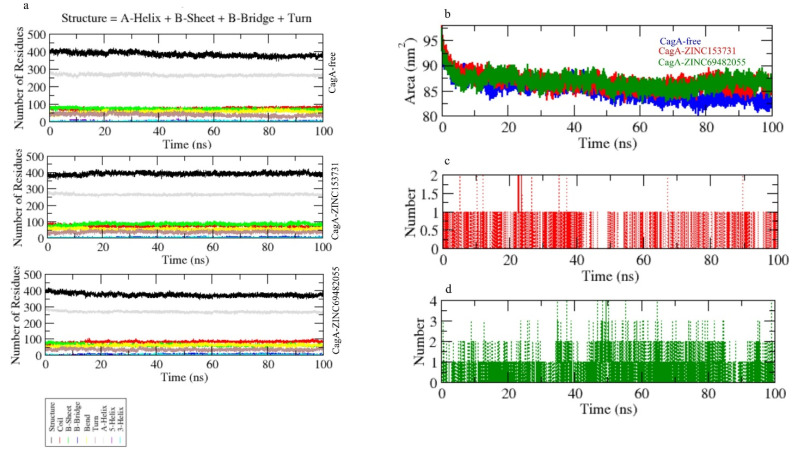
(**a**) Secondary structure changes upon ligand binding, (**b**–**d**) hydrogen bond analysis of complexes.

The secondary structural assignments in proteins such as -helix, -sheet, and turn were fragmented into specific residues at each time step. Because of enhancement in the fraction of coils and a decrease in -sheet, the average number of residues involved in secondary structure formation in complexes was lowered. In the case of CagA-ZINC69482055, the proportion of -sheet and -helix was observed to be considerably lower, and composition was changed upon binding with ZINC69482055 (Figure 7a). 

Hydrogen bonds are vital to the stability of the ligand–protein complex [38]. The hydrogen bonds paired were within 0.35 nm between protein and ligand. CagA-ZINC153731 and CagA-ZINC69482055 were estimated in a solvent environment during the 100 ns simulations to test the stability of docked complexes. CagA-ZINC69482055 strongly binds to the CagA pocket with 3–4 hydrogen bonds, whereas CagA-ZINC153731 binds to the CagA pocket with 1.5–2 hydrogen bonds and the least fluctuations (Figure 7c,d).

**Figure 8:**
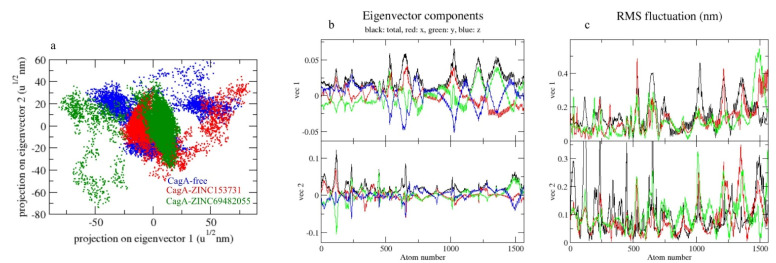
The 2D projection of complexes. (**a**) CagA-free, CagA-ZINC153731, and CagA-ZINC69482055, (**b**) Eigenvector components, (**c**) RMS fluctuation.

Principal component analysis depicts the overall expansion of a protein throughout simulations [39]. The sum of the eigenvalues is a measurement of the system’s overall motility, and it may be used to assess the flexibility of a protein under different conditions [40]. In 2D projections of trajectories on eigenvectors, the CagA-free and CagA- ZINC69482055 complexes showed overlap. The findings also showed that complexes binding to CagA cause a variation in atom positions (Figure 8a). 

**Figure 9:**
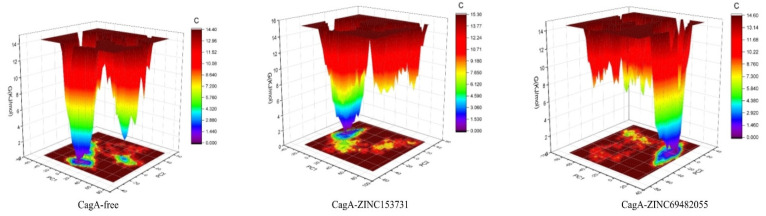
GFE landscape of complexes.

Gibbs’ free energy (GFE) landscape was also computed with Gromacs analysis commands and projections of their respective first (PC1) and second (PC2) eigenvectors. The comparable GFE contour map showed darker blue shades representing less energy. The global minima of CagA fluctuated during the simulations due to the complexes binding to CagA. CagA-free and CagA-ZINC153731 showed similar projections and CagA-ZINC69482055 showed different global minima, indicating that the ZINC69482055 compound formed a more stable complex in protein proximity (Figure 9). 

## 4. Conclusions

CagA inhibition is a novel approach for preventing the development of GC. For a long time, natural compounds have been the most important source of medicines for the treatment of many ailments. This study found that ZINC153731, ZINC69482055, and ZINC164387 efficiently bind to CagA protein and interact with the crucial CagA protein residue (Arg624). The study suggested that these compounds could be used as possible inhibitors of CagA protein in the fight against GC.

## Figures and Tables

**Table 1:** Physicochemical and drug-like properties analysis.

Descriptor/Properties	Value		
	ZINC153731	ZINC69482055	ZINC164387
Molecular Weight	178.187	358.434	267.275
Monoisotopic Mass	178.062995	358.178025	267.032953
Rotatable Bonds	2	1	0
Acceptors	3	5	2
Donors	1	1	1
cLogP	1.5561	3.0513	4.6289
cLogS	−1.831	−3.355	−4.672
Total Surface Area	76.271	264.17	177.93
Relative PSA	0.24733	0.23553	0.1627
Polar Surface Area	46.53	80.67	37.33
Drug-likeness	−4.3625	1.4711	−7.4682
Mutagenic	none	none	high
Tumorigenic	none	none	none
Irritant	none	none	none
Drug Score	0.4833717	0.7209172	0.1100568

**Table 2:** ADMET prediction of the top-scored natural compounds.

Property	Model Name		Predicted Value			Unit
			ZINC164387	ZINC69482055	ZINC153731	
Absorption	Water solubility		−5.452	−3.955	−1.944	log mol/L
	Caco2 permeability		1.527	1.292	1.189	log Papp in 10^−6^ cm/s
	Intestinal absorption		88.982	98.872	95.107	% Absorbed
	Skin permeability		−2.544	−4.244	−2.503	log Kp
	P-glycoprotein (P-gp) substrate		No	Yes	No	
	P-gp I inhibitor		No	No	No	
	P-gp II inhibitor		No	No	No	
Distribution	VDss (human)		0.504	0.004	−0.15	log L/kg
	Fraction unbound		0.018	0.267	0.396	Fu
	permeability	BBB	0.588	−0.133	0.125	log BB
		CNS	−1.264	−2.807	−1.937	log PS
Metabolism	substrate	CYP2D6	No	No	No	
		CYP3A4	Yes	No	No	
	inhibitor	CYP1A2	Yes	No	Yes	
		CYP2C19	Yes	No	No	
		CYP2C9	Yes	No	No	
		CYP2D6	No	No	No	
		CYP3A4	No	No	No	
Excretion	Total clearance		0.155	1.074	0.71	log mL/min/kg
	Renal OCT2 substrate		Yes	No	No	
Toxicity	AMES toxicity		No	No	No	
	Max. tolerated dose (human)		0.143	−0.361	0.931	log mg/kg/day
	inhibitor	hERG I	No	No	No	
		hERG II	No	No	No	
	LD50		2.54	2.592	1.833	mol/kg
	LOAEL		1.024	2.062	2.535	log mg/kg_bw/day
	Hepatotoxicity		No	No	No	
	Skin Sensitization		No	No	No	
	T. Pyriformis toxicity		2.26	0.49	0.8	log mM
	Minnow toxicity		0.033	1.667	1.168	

**Table 3:** BE of compounds with CagA protein.

S. No.	Compounds	2D Structure	Binding Energy (kcal/mol)	Inhibition Constant (µM)
**1**	ZINC153731	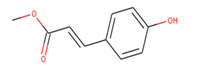	−11.53	10.9
**2**	ZINC69482055	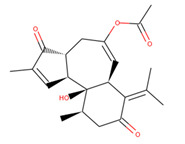	−10.67	13.32
**3**	ZINC164387	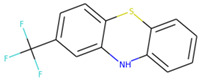	−9.21	18.56
**4**	DFMO *	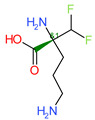	−7.25	28.85

* Control compound.

## Data Availability

The data presented in this study are available on request from the corresponding author.

## Sample Availability

Samples of the compounds are not available from the authors.
